# Duration of Breastfeeding and Its Correlates in Bangladesh

**DOI:** 10.3329/jhpn.v28i6.6608

**Published:** 2010-12

**Authors:** Shamima Akter, Md. Mizanur Rahman

**Affiliations:** Department of Population Science and Human Resource Development, University of Rajshahi, Rajshahi 6205, Bangladesh

**Keywords:** Breastfeeding, Parity, Sociodemographic factors, Bangladesh

## Abstract

The purpose of this study was to assess the duration of breastfeeding and the sociodemographic factors affecting it. Data for the study were drawn from the Bangladesh Demographic and Health Survey 2004. In total 5,364 mothers were included in the study. The life table and Cox's proportional hazards model were employed for the analysis of breastfeeding-related data, which showed that the average duration of breastfeeding was 31.9 months. Cox regression analysis revealed that the duration of breastfeeding was positively associated with maternal age, contraceptive-use, work status, and religion and was negatively associated with age at marriage, parity, delivery status, region, and maternal education. Younger mothers, having higher education, higher maternal parity, caesarean-section birth, being a Muslim, and mothers who have not used any contraceptive were associated with lower duration of breastfeeding. The findings suggest that health institutions can play a significant role in promoting breastfeeding in Bangladesh. Educational campaigns that stress the benefits of lactation are important strategies for encouraging mothers to breastfeed longer.

## INTRODUCTION

Breastfeeding plays a vital and influential role on the duration of amenorrhoea, child survival, and fertility, offering protection to an infant against early morbidity and mortality. Short-term risks of not breastfeeding include an increased risk of postpartum haemorrhage while long-term effects may include a higher risk of osteoporosis and breast and ovarian cancers (1). Breastmilk contains all types of nutrients required for an infant in right proportion and composition (2).

Longer and more frequent breastfeeding and maternal survival status ensure the survival of children (3–4). Results of studies on cessation of breastfeeding of children suggest that mothers who have lower education stop breastfeeding earlier than those with higher education (5–7). Other factors that also relate to the duration of breastfeeding are present age of mothers and socioeconomic status (7–12). Younger mothers are most likely to terminate breastfeeding early compared to older counterparts (13–15).

The importance of breastfeeding in regulating individual and social fertility has been a matter of general interest for many years because it tends to increase the average birth interval and, therefore, to reduce women's fertility over their life span, especially in societies where the use of contraceptive methods is not widespread. Recent reviews and meta-analyses conclude that breastfeeding constitutes a small but consistent protective effect against obesity or higher values of body mass index (BMI) in children (16–19).

The propensity to breastfeed is not only of importance with regard to the beneficial effects on individuals but is also of concern as an indicator of health behaviour relating to social conditions. A large body of research supports an association between the socioeconomic status and the health and development of children (20–24) but this concept has rarely been studied in relation to the duration of breastfeeding in the context of Bangladesh. The aim of this paper was to estimate the duration of breastfeeding and also to explore the sociodemographic determinants of the duration of breastfeeding in Bangladesh.

## MATERIALS AND METHODS

Data for the study were drawn from the Bangladesh Demographic and Health Survey (BDHS) 2004. Information was collected on education, age, reproductive behaviour, availability of family-planning supplies and services, breastfeeding, child health, and maternal status. The survey considered ever-married women of reproductive age as eligible for interview. A sample of 10,500 households was selected from which 11,444 women were interviewed. In total, 5,364 mothers provided information on the duration of breastfeeding for their last-born child at the time of interview. The maximum number of months of breastfeeding recorded in the survey was 60 but our study included the duration of breastfeeding up to 48 months and ignored the remaining months as outlier.

### Measurement of variables

#### Dependent variable

The duration of breastfeeding the last child of the respondent was the dependent variable which was calculated as the number of months that the mother reports having breastfed the child.

#### Independent variables

The demographic variables included age of mother, age-at-marriage, sex of child, parity, contraceptive-use, and delivery status.

For analysis of data, the age of the respondent was categorized into three broad groups: ≤24 years, 25–34 years, and 35 years and over. The age-at-marriage was classified into four categories: ≤14 years, 15–19 years, 20–24 years, and 25 years and over. The parity of mother in the sample was divided into four major groups: 1, 2, 3–5, and ≥6. The category relating to contraceptive-use was dichotomous: not used and used. The delivery status was assessed as either normal or by caesarean section.

The socioeconomic variables included place of residence, region of residence/administrative division, the highest level of education of the respondent and the husband, and occupation of the respondent.

Education of the respondent is the highest level of schooling attained, measured as no education, primary, secondary, and higher; education of her spouse was also measured as no education, primary, secondary, and higher. Occupation of the respondent was also measured as a categorical variable: not working and professional/administrative, clerical/sales, skilled/unskilled manual, domestic, and others.

### Statistical analysis

Bivariate and multivariate statistical techniques were used for studying the predictor variable—duration of breastfeeding—in relation to the explanatory variables. The variable—duration of breastfeeding—was coded as 0–12 months, 12–24 months, 24–36 months, and 36–48 months. The association of the duration of breastfeeding with all the independent variables in the study was first checked by the chi-square statistic. Life-table analysis provides a good understanding of breastfeeding behaviour over time. The factors affecting the duration of breastfeeding were investigated in a regression using Cox's proportional hazard model (25). This model was used for determining the covariates that were significantly associated with the duration of breastfeeding. The data obtained were analyzed using the SPSS software (version 10.0 (26), Statistica 5.0, and Excel.

### Life-table technique

A life-table can be constructed by pooling completed and censored cases of breastfeeding (27–28). The completed observations were those in which breastfeeding was stopped, and the exact duration of breastfeeding was known. Censored observations were those in which the child was still being breastfed at the time of survey. The mean duration of breastfeeding was used as the summary measures.

### Cox's proportional hazard model

Survival analysis technique was used in analyzing breastfeeding-related data. The survival analysis technique adjusts for truncation bias by incorporating both complete and incomplete segments of histories in the analysis of breastfeeding-related data (some mothers might be continuing to breastfeed at the time of the survey). The Cox's proportional hazards (PH) model may be viewed as a multivariate life-table but unlike other regression techniques, this method uses censored data and, thus, controls for truncation bias (29). Descriptive statistics and the individual effects of Cox regression analysis for each variable are given to provide a general overview of the covariates in the analysis (30). The hazard function at time-point *t* (stopping or termination of breastfeeding), denoted λ (t,z), by is expressed as: λ (t,z) = λ_0_(*t*).exp ∑ X_i_ ß_i_, where λ(t,z) is the hazard rate at time *t*, λ_0_(*t*) is the baseline hazard function of t, *ß_i_* is a vector of coefficients, and X_i_ is a vector of covariates. It is assumed in this model that: (a) there is a hazard or risk of occurrence of the event of interest (in this case, the termination of breastfeeding) at each time *t*, and this is applicable to all members of the population; (b) at each time *t*, the respondents at one level of a given subgroup experience a hazard proportional to the reference category; the models are a function of time and regressor variables; and (c) there will only be one set of coefficients. The hazard ratio (odds ratio) for breastfeeding and its 95% confidence interval (CI) were calculated for the sociodemographic factors associated with breastfeeding.

## RESULTS

The figure shows the overall pattern of breastfeeding in Bangladesh. The survival curve represents the probability of mothers who continued to breastfeed at any given time. During the first month of life, the maximum (87%) probability of continuing breastfeeding was observed, which decreased dramatically after two years of age.

**Fig.**
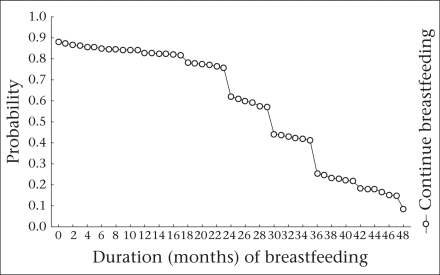
Survival function for women who were continuing breastfeeding at different durations of breastfeeding, Bangladesh

The life-table analysis and its related measure (mean) for the duration of breastfeeding by various covariates of mother and child are shown in Table 1. Bivariate analysis revealed significant differences in the duration of breastfeeding relating to place of residence, administrative division, delivery status, contraceptive-use, education of mother, education of husband, parity, and religion.

**Table 1.** Mean duration of breastfeeding by different sociodemographic characteristics based on life-table technique, Bangladesh, 2004

Characteristics	No.	Mean	p value
Age (years) of mothers			
≤24	2,659	32.69	
25–34	2,217	31.42	
35 and above	579	31.63	0.128
Age-at-marriage (years)			
≤14	2,745	32.70	
15–19	2,337	31.49	
20–24	323	30.53	
25+	50	28.85	0.007
Sex of previous child			
Male	2,774	32.00	
Female	2,681	31.99	0.879
Parity			
1	1,529	32.42	
2	1,375	32.04	
3–5	1,967	32.13	
6 and above	584	31.82	0.005
Contraceptive-use			
Not using	917	31.47	
Currently using	4,538	32.13	0.000
Delivery status			
Normal	5,189	32.13	
Caesarian	266	30.12	0.000
Place of residence			
Urban	1,707	30.51	
Rural	3,749	32.71	0.000
Division/region			
Barisal	619	32.83	
Chittagong	1,129	27.78	
Dhaka	1,224	32.25	
Khulna	725	33.79	
Rajshahi	1,101	33.72	
Sylhet	657	32.16	0.000
Education of mothers			
No education	1,924	32.60	
Primary	1,675	32.96	
Secondary	1,524	30.71	
Higher	338	29.90	0.013
Work status of respondents			
Working	998	32.25	
Not-working	4,457	31.90	0.218
Education of husband			
No education	2,035	33.01	
Primary	1,466	32.35	
Secondary and higher	1,954	30.65	0.004
Religion			
Muslim	4,962	31.53	
Non-Muslim	493	36.13	0.062
Bangladesh	5,455	31.99	-

The mean duration of breastfeeding in Bangladesh for surviving children is 31.9 months. It is comparatively higher among younger mothers (<25 years) than older mothers (≥35 years). In this study, the mean duration of breastfeeding was 32.67 months for mothers of younger age-group, 31.4 months for middle-aged mothers, and 31.6 months for older mothers. Mothers who were married at an early age had a longer duration of breastfeeding than those who were married in older age. Mothers of single parity breastfed their children, on average, for 32.4 months; mothers of 2 parity breastfed for 32.0 months; and those with 3–5 parity breastfed for 32.1 months. This decreased to 31.8 months for those who had six and more children. The average duration of breastfeeding was 31.4 months for mothers who were not using any contraception compared to 32.1 months for those who were using contraception. The mean duration of breastfeeding was comparatively lower among mothers who gave birth by caesarean section (30.1 months) compared to mothers giving birth vaginally (32.1 months).

The urban mothers breastfed their children for a relatively-shorter duration than did the rural mothers. Among the six administrative divisions of Bangladesh, the duration of breastfeeding was the lowest in Chittagong division (27.78 months). The non-Muslim mothers breastfed for a longer duration (36.13 months) than the Muslim mothers (31.53 months).

Table 2 presents the results of the proportionality hazards model for the duration of breastfeeding. Age of mother, age-at-marriage, parity, contraceptive-use, delivery status, region, religion, education and occupation of mother were statistically significant. The odds of stopping breastfeeding for older mothers were lower than their younger counterparts. Women who married at an early age (≤14 years) had a lower risk of stopping breastfeeding compared to women who married at an older age (25 years and over). Increased parity was associated with increase in the risk of cessation of breastfeeding. Normal delivery was associated with a 23% less likelihood of terminating breastfeeding compared to birth by caesarean section. Similarly, the use of contraceptives had a lower risk of stopping breastfeeding. The mothers of Chittagong and Sylhet divisions were more likely to terminate breastfeeding early compared to the mothers in other divisions. The Muslim mothers had 1.3 times higher risk of stopping breastfeeding than their non-Muslim peers. The risk of cessation of breastfeeding increased with increasing maternal education. Mothers not working were 1.16 times more likely to stop breastfeeding than working mothers.

**Table 2.** Cox's proportional hazard model estimates of relative risk of sociodemographic characteristics on cessation of breastfeeding, Bangladesh, 2004

Explanatory variable	Odds ratio	95% CI
Age (years) of mothers		
≤24	2.114*	1.851–2.414
25–34	1.386*	1.239–1.551
35+	1.000	
Age-at-marriage (years)		
≤14	0.593*	0.439–0.801
15–19	0.634*	0.471–0.853
20–24	0.699**	0.513–0.954
25+	1.000	
Sex of child		
Male	0.971	0.919–1.026
Female	1.000	
Parity		
1	0.632*	0.545–0.733
2	0.691*	0.603–0.790
3–5	0.991	0.813–1.022
6+	1.000	
Contraceptives-use		
No used	1.387*	1.276–1.507
Used	1.000	
Delivery status		
Normal	0.767*	0.669–0.881
Caesarean	1.000	
Place of residence		
Urban	1.007	0.947–1.072
Rural	1.000	
Division/region		
Barisal	0.874**	0.776–0.985
Chittagong	1.133**	1.021–1.256
Dhaka	0.903***	0.813–1.003
Khulna	0.929	0.825–1.045
Rajshahi	0.861*	0.771–0.961
Sylhet	1.000	
Religion		
Muslim	1.289*	1.169–1.422
Non-Muslim	1.000	
Educational level		
No-education	0.790*	0.680–0.917
Primary	0.820*	0.712–0.943
Secondary	0.891**	0.782–1.015
Higher	1.000	
Work status		
Do not work	1.161*	1.079–1.249
Work	–	
Education of husband		
No education	0.997	0.920–1.079
Primary	1.002	0.927–1.083
Secondary and higher	1.000	
Log-likelihood	77,191.721	
Model chi-square	405.96	
Degrees of freedom	24	
p value	0.000	

*p<0.01;

**p<0.05;

***p<0.10, significant variables in the model;

CI=Confidence interval

## DISCUSSION

The study examined the socioeconomic and demographic determinants of breastfeeding in Bangladesh. Breastfeeding is virtually universal (98.3%) and prolonged in Bangladesh. Past studies in Bangladesh found the mean duration of breastfeeding to be 26.4–28.9 months (30–33). It seems that the duration of breastfeeding in Bangladesh is gradually increasing.

Increasing maternal age and parity can lead to breastfeeding of a shorter duration. Higher parity leads to shorter birth intervals and, hence, shorter time available for breastfeeding. It is also well-established that parity is closely related to maternal age (31). An older woman is more likely to have a greater number of children; hence, the demand on her time is considerable which may lead to early termination of breastfeeding. Poor nutritional status, particularly among older women, can diminish the capacity and the fat and vitamin content of breastmilk. The result is that not enough breastmilk will be provided to the infant, thus, hastening early termination of breastfeeding. In this study, mothers with 1, 2, and 3–5 parity were 37%, 31%, and 1% less likely to terminate breastfeeding than mothers with ≥6 parity, suggesting that an increase in parity is associated with a decrease in probability of terminating breastfeeding. Giving births to too many children might have caused physical complications and weakness to these mothers who were unable to breastfeed their children. Women aged ≤14 years, 15–19 years, and 20–24 years were less likely to terminate breastfeeding than women who were married at ≥25 years. The higher risk of terminating breastfeeding in mothers with first-born babies might result from two reasons. First, they belonged to younger age-group, and second, they neither had breastfeeding experience nor they feel comfortable in breastfeeding their children. The use of contraceptives plays a role in lengthening inter-pregnancy interval, and thus, mothers have time to breastfeed their children, consequently reducing the risk of termination.

Region of residence of the respondents had a significant effect on the risk of termination of breastfeeding, for example, mothers from Chittagong were more likely to terminate breastfeeding than those from Sylhet. This is in agreement with the results of previous studies (10–11, 30). One of the key determinants of the decline in breastfeeding in Bangladesh is the increasing level of education of mothers, a factor which plays a role in the adoption of modern ideas and which usually leads to the abandonment of traditional practices regarding childcare. A similar trend that higher education is associated with shorter duration of breastfeeding was also observed in some earlier studies in Bangladesh (10–11) and in other developing countries (34), although the scenario in industrialized countries, such as Denmark, appears to be the opposite (35). Results of a study suggest that education is a proxy for socioeconomic status, which could be related to exposure to advertisements and the capability to buy infant formula (36). The probability of terminating breastfeeding by Muslim mothers was higher compared to non-Muslim mothers (Hindu, Christian, and Buddhist). Working women breastfed for a slightly longer duration compared to the non-working women, which is consistent with the findings of other studies (30, 32). Women in Bangladesh are involved in traditional or informal work (agricultural activities, domestic work, jobs in cottage industries, and small-scale marketing, or as labourer), especially in the rural areas, and have more flexible schedules, and this allows them to nurse their infants more often, thus maintaining longer periods of lactation.

The results of the present study indicate that the breastfeeding-promotion programme in Bangladesh should address mothers with higher education, those who have higher parity, give birth by caesarean section, and those living in urban areas and Chittagong since these mothers tend to breastfeed their children for a relatively-shorter period of time.

## ACKNOWLEDGEMENTS

The authors thank the anonymous reviewers of this journal for providing insightful comments and suggestions for revision.
